# Comparison of the Rat and Human Dorsal Root Ganglion Proteome

**DOI:** 10.1038/s41598-018-31189-9

**Published:** 2018-09-07

**Authors:** Adam G. Schwaid, Alicja Krasowka-Zoladek, An Chi, Ivan Cornella-Taracido

**Affiliations:** 1 MRL, Merck & Co., Inc., Boston, MA 02115 USA; 2 MRL, Merck & Co., Inc., West Point, PA 19846 USA; 3 Present Address: Cedilla Therapeutics, Cambridge, MA 02139 USA

## Abstract

Dorsal root ganglion (DRG) are a key tissue in the nervous system that have a role in neurological disease, particularly pain. Despite the importance of this tissue, the proteome of DRG is poorly understood, and it is unknown whether the proteome varies between organisms or different DRG along the spine. Therefore, we profiled the proteome of human and rat DRG. We identified 5,245 proteins in human DRG and 4959 proteins in rat DRG. Across species the proteome is largely conserved with some notable differences. While the most abundant proteins in both rat and human DRG played a role in extracellular functions and myelin sheeth, proteins detected only in humans mapped to roles in immune function whereas those detected only in rat mapped to roles in localization and transport. The DRG proteome between human T11 and L2 vertebrae was nearly identical indicating DRG from different vertebrae are representative of one another. Finally, we asked if this data could be used to enhance translatability by identifying mechanisms that modulate cellular phenotypes representative of pain in different species. Based on our data we tested and discovered that MAP4K4 inhibitor treatment increased neurite outgrowth in rat DRG as in human SH-SY5Y cells.

## Introduction

A key factor in drug discovery success is translatability between preclinical models and human disease. Without this ability progressing drug discovery programs is difficult as there is no practical way to test compounds or mechanisms *in vivo* prior to the clinic. This is a particular challenge in pain biology and neuroscience in general since many of these models can be plagued with poor translatability^1,2^. We envision that proteomics as one of several molecular pheonotyping – omics tools can help navigate the problem of translatability by allowing us to prioritize mechanisms or targets that have similar expression in humans and model systems. While protein expression may not correlate to protein function or activation status, proteomics studies can characterize differences between one tissue and another and can lead to understanding at a mechanistic level of the cellular markers and pathways that may vary from tissue to tissue. For example, detailed proteomics studies in brain have been carried out to gain greater insights into the functioning of the central nervous system (CNS)^3^. Dorsal root ganglion (DRG), are an important part of the peripheral nervous system^4,5^.

DRG are believed to have a key role in sensation of pain and transmission of signals to the brain^4,5^. DRG are commonly used to study pain in models that may measure one of several different phenomena. Electrophysiology and measurements of conductance and gating are one type of model frequently related to pain. For instance, studies focusing on Nav1.7, a promising target for pain, utilize assays measuring current amplitude and polarization in DRG^6,7^. Neurite outgrowth using DRG explants have also been used to model pain; Examples include studies focusing on the roles of Neurite Growth Factor (NGF) and others^8–10^. Despite the important role of DRG in human physiology, the proteome of this tissue has not been comprehensively profiled. Several papers have searched for proteins changing in rodent DRG between healthy and injured animals using 2D-gel electrophoresis coupled to mass spectrometry. These studies expanded our undestanding of proteins involved in pain, but were only able to identify a small number of proteins^11,12^. The most comprehensive protein profiling experiment published to date analyzed only 2526 proteins in mice and focused on membrane proteins^13^. Moreover, no large scale proteomics experiments have studied the DRG proteome in either rats or humans. We conducted proteomic studies in rat and human DRG in order to better navigate translation between these two systems. Based on this data, we predicted and observed that treatment of rat DRG with a MAP4K4 inhibitor would increase neurite outgrowth, similar to effects observed in human SH-SY5Y cells.

## Results

### Human DRG proteome

**Figure 1:**
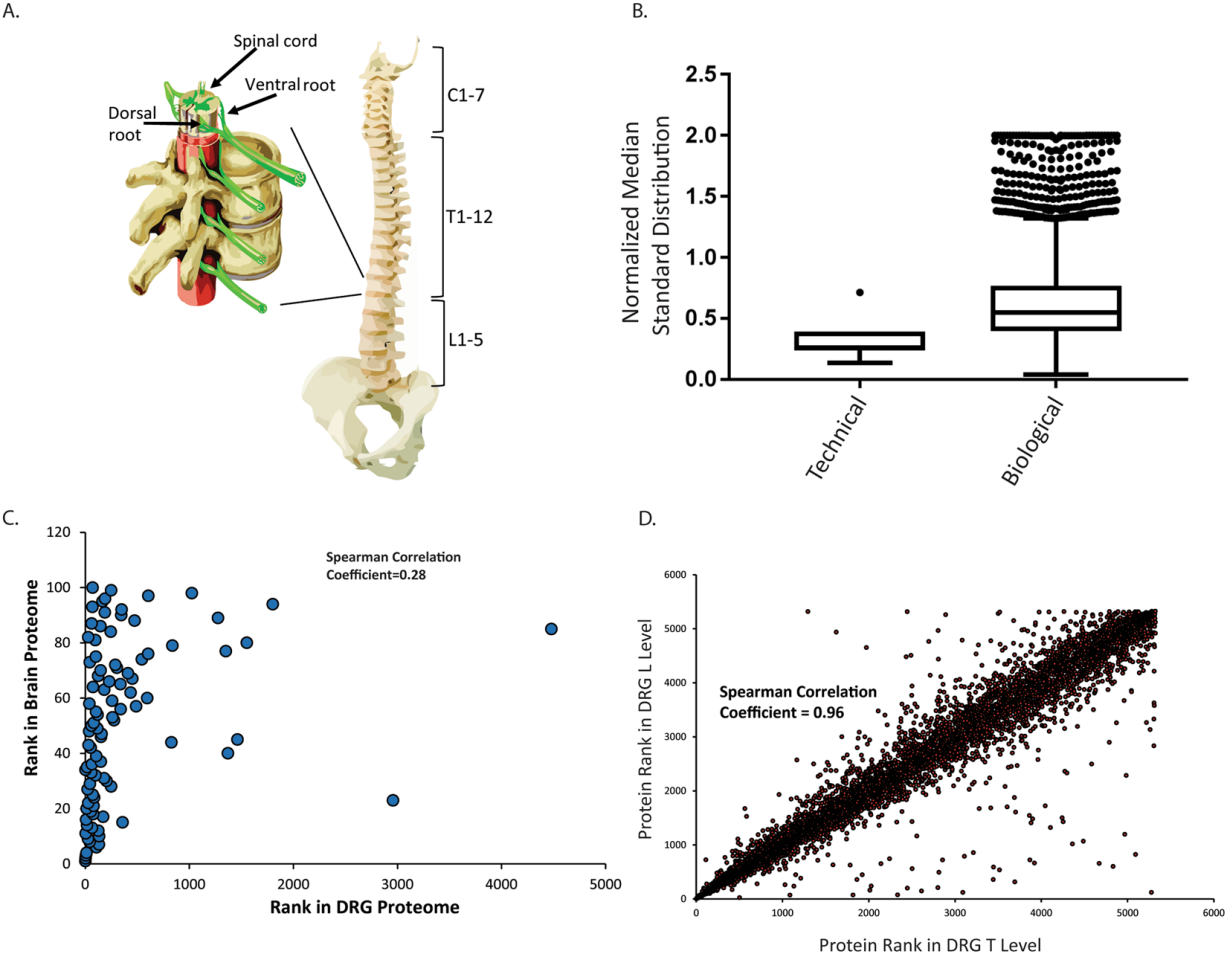
(**A**) Diagram of the human spine and location of vertebrae levels marked. Insert shows enlarged vertebrae with dorsal root highlighted. (**B**) Median standard deviation of technical and biological replicates in human DRG. The normalized median standard deviation of technical replicates were calculated by normalizing IBAQ values of each technical replicate from a biological sample to the average IBAQ value from both technical replicates of that biological sample. Normalized technical replicate values were used to calculate a standard deviation and the median standard deviation was found. The median normalized standard deviation of technical replicates from each biological replicates was plotted. Each data point is the median standard deviation of a set of technical replicates from a biological replicate. Median standard deviation of biological replicates was calculated by normalizing the intensity of a protein in one biological replicate to the average intensity of that protein across biological replicates. Standard deviations were computed based on normalized values and the median value was plotted. Each data point is a the median standard deviation of a protein. Data are plotted as a Tukey Box and whisker plot. (**C**) Comparison the rank order of 100 most abundant proteins in human brain to human DRG proteome shows no correlation (Spearman Correlation Coefficient 0.28). (**D**) Protein abundance between T11 and L2 levels of human DRG are strongly correlated (Spearman Correlation Coefficient 0.96).

In order to understand the human DRG proteome we conducted in-depth shotgun proteomics on DRG tissue harvested from the T11 and L2 vertebrae of 4 human donors immediately post mortem (see supplementary methods). After filtering for proteins that were detected in at least two individuals we identified 5,245 proteins (Supplementary Table 1). Two technical replicates were performed per sample. Technical variance was low, and protein abundance had a median standard deviation of 0.26. On the other hand, protein abundance varied somewhat more between biological replicates and had a median standard deviation of 0.55 (Fig. 1). In combination with the low technical variance of the experiments this indicates protein abundance between humans does show a relevant level of variation that should be considered during experimental design.

First we sought to understand how DRG varied from other nervous system tissue such as brain. To answer this question we compared our human DRG data to proteomics data from human cerebral cortex reported by Wilhelm *et al*.^14^. Since many proteins are expressed at some level in most tissues the top expressed proteins in a given tissue can be considered characteristic of that tissue. Therefore, we compared the abundance of the one hundred most abundant proteins in brain to their abundance in DRG and found that most (93) of these proteins are also detected in human DRG. However, only 37 of these proteins were amongst the one hundred most abundant in DRG. When we compared the ranked abundance of proteins that were detected we found no correlation (Spearman’s Correlation, R^2^ 0.28). This indicates that these two nervous system tissues have distinct proteomes (Fig. 1). Therefore, studies performed in brain should not be expected to translate to DRG and vice versa.

DRG are neuronal sensory bodies located outside the blood-brain barrier along the spine and integrate signals from the body. Two DRG are present per vertebrae and DRG can vary morphologically along the spine. One question one may ask when studying pathways or phenotypes in human DRG is from which vertebrae should DRG be examined in order to model a particular process. Therefore, we next asked if there were any quantitative differences in the proteome between DRG from the T11 and L2 vertebrae. However, Label Free Quantitation measurements (LFQ) calculated with maxquant, which uses the extracted ion intensity of peptides and applies a delayed normalization, indicated no substantial enrichment of any protein in either group^15^. Althougth, some outliers are apparent on the correlation plot the differences in these particular proteins is not statistically significant between the T and L levels. Furthermore, the ranked abundance of proteins correlated extremely well between both levels of DRG with a Spearman Correlation Coefficient of 0.96 (Fig. 1). In their naïve state there is no difference in the proteome or protein abundance in DRG from the T and L vertebrae levels. These results indicate that DRG, at least from the levels examined in this study, can be used interchangably when studying DRG biology.

### Rat DRG Proteome

**Figure 2:**
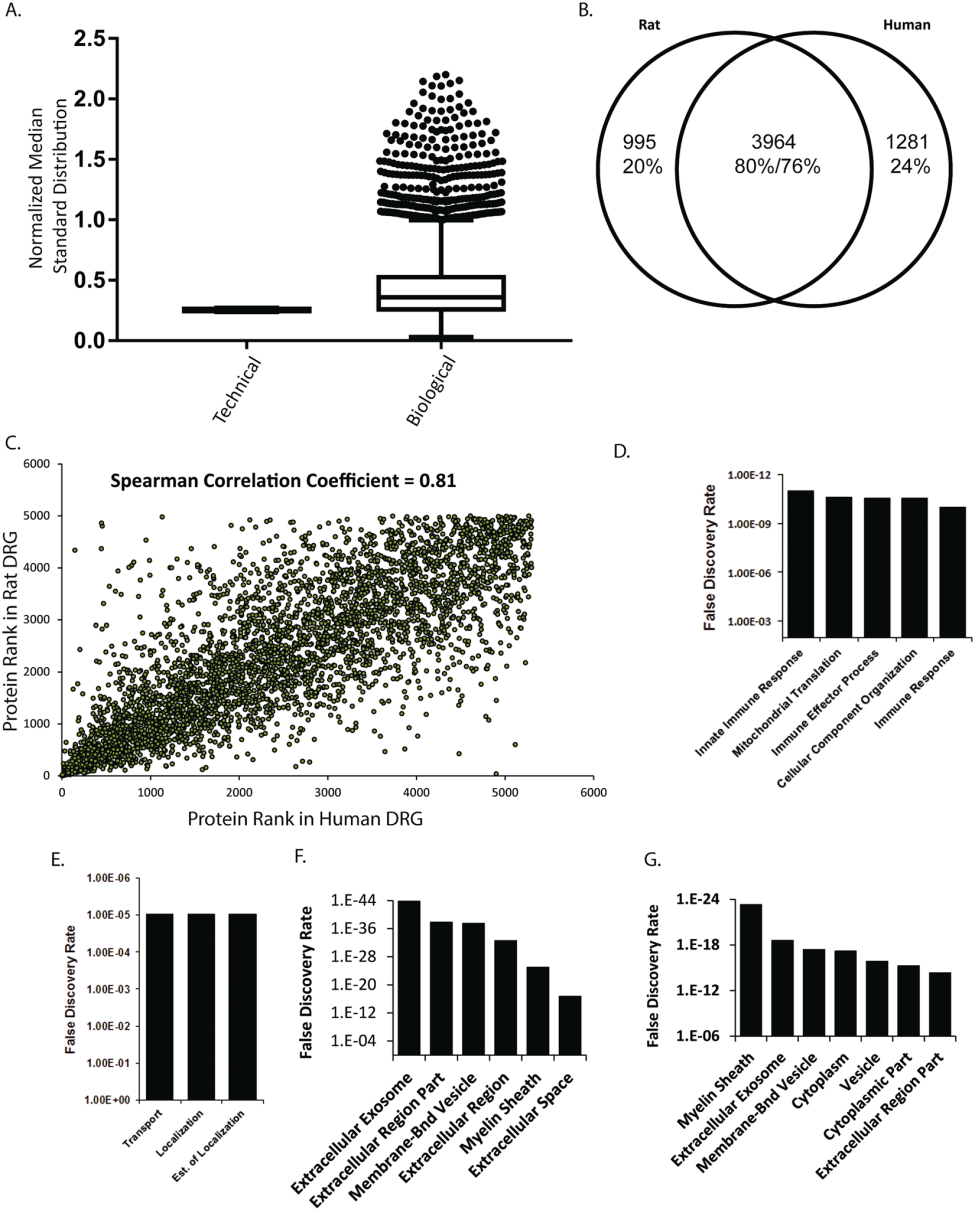
(**A**) Median standard deviation of technical and biological replicates in rat DRG. The normalized median standard deviation of technical replicates were calculated by normalizing IBAQ values of each technical replicate from a biological sample to the average IBAQ value from both technical replicates from a biological sample. Normalized technical replicate values were used to calculate a standard deviation and the median standard deviation was found. The median normalized standard deviation of technical replicates from each biological replicates was plotted. Each data point is the median standard deviation of a set of technical replicates from a biological replicate. Median standard deviation of biological replicates was calculated by normalizing the intensity of a protein in one biological replicate to the average intensity of that protein across biological replicates. Standard deviations were computed based on normalized values and the median value was plotted. Each data point is a the median standard deviation of a protein. Data are plotted as a Tukey Box and whisker plot. (**B**) Venn-Diagram of share and unique proteins between rat and human DRG. Most proteins are present in DRG of both species however a subset are unique to each. (**C**) Correlation of ranked protein abundance between rat and human DRG showed a high degree of similarity (Spearman correlation coefficient 0.81). (**D**) Gene Ontology analysis of proteins detected in human but not rat DRG when mapping to biological processes gene sets. (**E**) Gene Ontology analysis of proteins detected in rat but not human DRG when mapping to biological processes gene sets. (**F**) Gene Ontology analysis of top 100 most abundant proteins in human or (**G**) rat DRG mapping to cellular component gene sets.

We sought to understand if DRG from rat could be used as surrogates for DRG from human. Proteomics in rat DRG identified 4,959 proteins from 5 rats with 2 technical replicates per sample (Supplementary Table 2). Technical variance in protein abundance was low as in our measurements in human DRG (median standard deviation 0.25). Whereas biological variance in protein abundace was 0.36 (Fig. 2). Based on the higher biological variability in humans than rats it should be expected that studies in humans or human samples will need substantially more samples to reach the same level of powering achieved with a given number of rats.

We compared the ranked abundance of proteins in the rat DRG to the ranked gene expression measured by Sapio *et al*. (Supplementary Fig. 1)^16^. The Spearman Correlation between these two datasets was 0.39 consistent with previous observations of protein and mRNA abundance correlation^17^. Next we investigated the similarity in the proteins detected between rat and human DRG. Of the 4,959 proteins detected in rat DRG, 3,964 of them were also detected in human DRG (Fig. 2). In large, the proteins present in one species are also present in the other, supporting that in general rat DRG can be used as models for human tissue. We also compared the relative abundance of proteins in human and rat DRG. Since the same protein may vary in sequence or length rather than directly comparing IBAQ, an intensity measurement that uses the sum of all peptide peak intensities matched to a protein divided by the number of theoretically observable peptides, or LFQ values we compared the abundance ranking of each protein in its own proteome to its abundance ranking in the other species^18^. This showed a reasonable correlation between protein rankings in human and rat DRG (Spearman Correlation coefficient = 0.81, Fig. 2). Of the proteins that were expressed in human, but not rat DRG these were enriched in pathways that related to immune function (Fig. 2 and Supplementary Table 3), which is sensible considering the available literature indicating substantial differences between the human and rodent immune system^19,20^. Proteins only detected in rat DRG mapped to pathways involved in transport and localization (Fig. 2 and Supplementary Table 4). We next sought to characterize the functional roles of the most abundant proteins in human and rat DRG. Using Gene Ontology Cellular Component analysis we found that the 100 most abundant proteins in human or rat DRG matched predominantly to roles in extracellular trafficking and the myelin sheath (Fig. 2). This is consistent with the previous characterization of this tissue being predominantly involved in integration of extracellular stimuli and signal transmission. Furthermore, it builds confidence that in many cases rat and human DRG can be used interchangeably when the question being studied is focused on these phenomena.

### Comparison of Human and Rat DRG proteome

**Figure 3:**
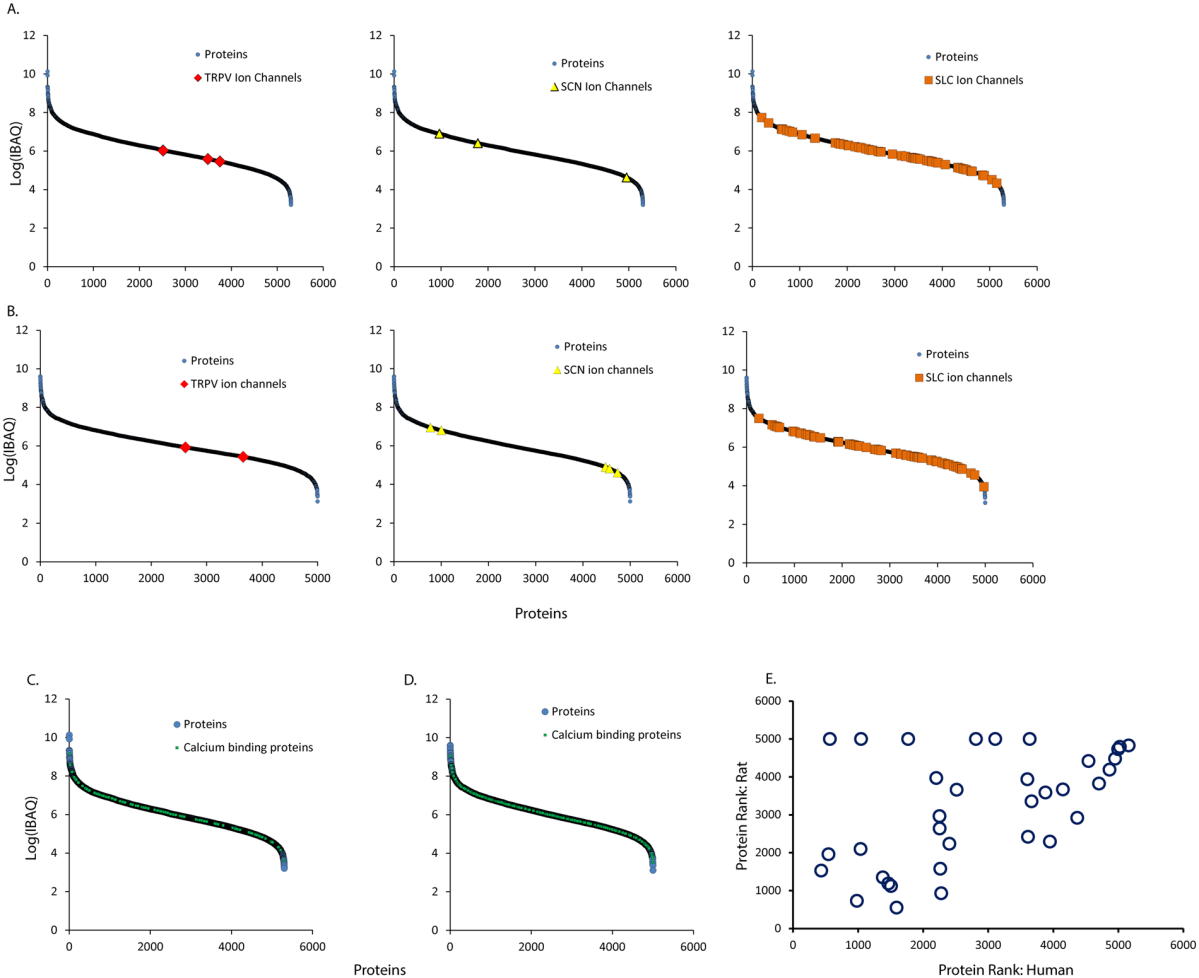
(**A**) Relative abundance of ion channel family members in human DRG. Proteins are plotted by ranked abundance from left to right. Ion channel family members are highlighted. (**B**) Relative abundance of ion channel family members in rat DRG. Proteins are plotted by ranked abundance from left to right. Ion channel family members are highlighted. (**C**) Relative abundance of calcium binding proteins in human DRG. Proteins are plotted by ranked abundance from left to right. Calcium binding proteins are highlighted. (**D**) Relative abundance of calcium binding proteins in rat DRG. Proteins are plotted by ranked abundance from left to right. Ion channel family members are highlighted. (**E**) Ranked protein abundance of proteins with annotated relevance to pain in human or rat (Table 1). Proteins correlated well with a Spearman correlation coefficient of 0.50. A subset of proteins appeared to be much more abundant in human than rat.

Often DRG are scrutinized in studies of pain due to their key role in sensing and transmitting signals to the brain. Ion channels are believed to be a key class of proteins involved in the sensation of pain^21^. Therefore we highlighted the relative abundance of ion channels we detected in DRG. Our analysis of the human DRG proteome covered seven orders of magnitude, and most detected ion channels fell into the bottom half of protein abundance, though there were some highly expressed ion channels (Fig. 3). Rat DRG showed a similar pattern. Likewise, calcium flux is implicated in pain sensation so calcium binding proteins are another class of proteins worthy of scrutiny^21^. We found the abundance of calcium binding proteins to be evenly distributed across the proteome with both very high abundance and low abundance proteins present. This pattern was consistent in rat and human. These data show that in many cases ion channels or calcium binding proteins that are believed to be important in man can be productively studied in rat or *vice versa*. However, care should be taken when investigation is focused on a particular protein to verify that this individual protein and its network of interactors shows similar expression levels in human and rat (Supplementary Tables 1 and 2).

Additionally, we conducted a focused analysis on a subset of proteins that are acknowledged to be especially relevant to pain^21^. These include Nerve Growth Factor Receptor (NGFR), TRPV1, and Nav ion channels; proteins involved in prostaglandin synthesis or prostaglandin receptors; gaba-aminobutyric acid receptor associated proteins; calcium dependent ion channels and kinases; purinogenic receptors; kinases involved intracellular signal transduction from membrane receptors, such as PI3Ks, PKA, and PKC; and additional proteins (Table 1). Overall the rank order of each protein in its proteome correlated well across species with a few notable exceptions (Spearman Correlation Coefficient 0.5, Fig. 3E); NGFR and TRPV1 were more abundant in human than rat whereas CAMK1 and CAMK2G were more abundant in rat than human. Some proteins were not detected in one species while they were detected in the other. In some cases this may be due to the low abundance of a particular protein, the limit of detection of the instrument, and technical limitations of stoichastic sampling. For example, Nav1.7 is detected as the 4695 out of 4959 most abundant protein in rat DRG while it is not detected in human DRG. In light of this protein’s low abundance and other reports that detect Nav1.7 in human DRG it is likely that it is present in human DRG just at a very low level. In other cases, some proteins that are expressed at high levels in human DRG are not detected in rat DRG-PTGDS is the 568th most abundant in human DRG but was not detected in rat. Given the abundance of this protein in human DRG this indicates a substantial decrease in PTGDS protein abundance in rat. Interestingly, prostaglandin related proteins in general did not show any correlation in protein abundance between human and rat.

**Table 1:** The ranked abundance of proteins with annotated roles in pain in rat or human DRG.

Gene Name	Ranked Abundance: Human	Ranked Abundance: Rat
NGFR	437	1522
PTGES3	547	1944
PTGDS	570	ND
GABARAPL2	989	727
PRKACB	1046	2084
CAMK2B	1054	ND
PRKACA	1387	1344
CAMK2D	1473	1182
PRKAR2B	1513	1117
CAMK2G	1601	548
GABARAP	1777	ND
PIK3CA	2201	3940
PTGES2	2250	2944
CACNA2D1	2252	2623
P2RX7	2260	1569
PTGR2	2274	924
CAMK2A	2400	2221
TRPV1	2511	3630
CAMK1D	2802	ND
P2RY12	3095	ND
PIK3R1	3577	3906
CAMK1	3585	2403
PTGS1	3612	ND
FAAH	3641	3332
PRKCD	3851	3561
PRKAR1B	3919	2279
PRKCI	4115	3638
PRKCE	4330	2899
PIK3C3	4500	4381
PIK3R4	4660	3792
IL6ST	4821	4155
SCN11A	4905	4442
CACNA2D2	4975	4753
PIK3C2A	5108	4790
SCN9A	ND	4695

**Figure 4:**
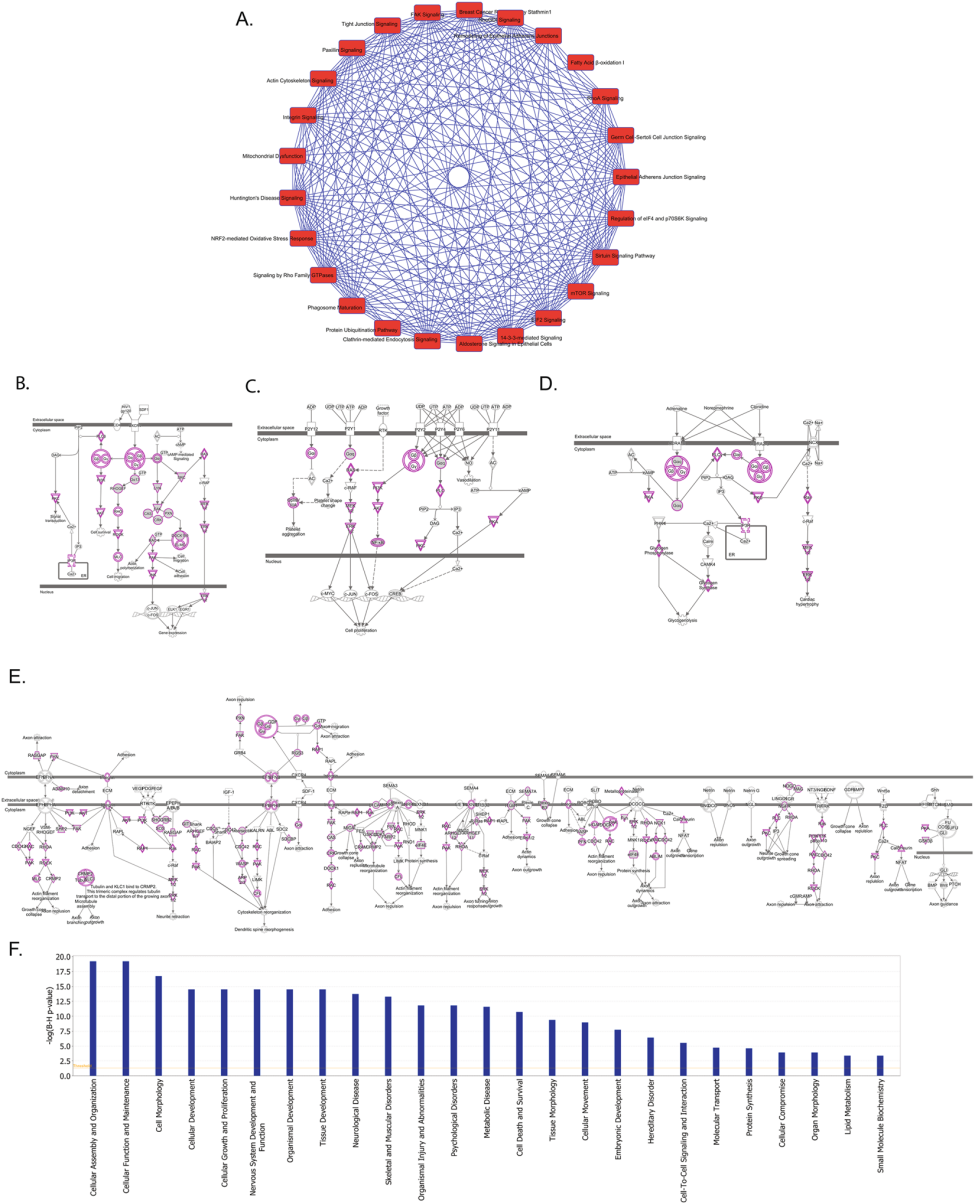
(**A**) Top ranked pathways represented by proteins detected in rat and human DRG. Blue lines between pathways represent overlap or interconnections. (**B**–**E**) Diagrams of conserved pathways with proteins detected in both species highlighted in purple. B. CRXC4 signaling C. P2Y signaling. (**D**) Adregenergic signaling. (**E**) Axonal guidance signaling. (**F**) Categories of the top most conserved cellular functions.

**Figure 5:**
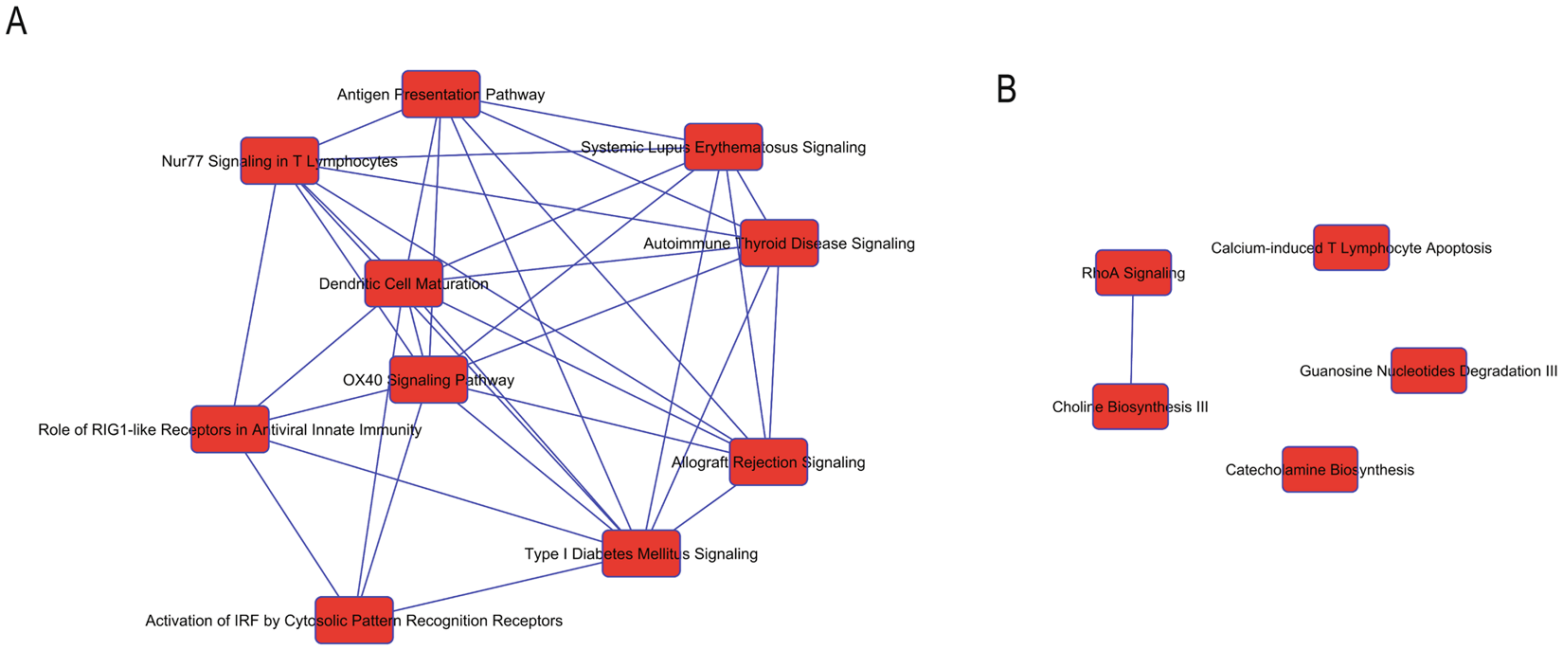
(**A**) Pathways represented by proteins detected only in human tissues. Blue lines represent overlap and connections between pathways. (**B**) Pathways represented by proteins detected only rat tissues. Blue lines represent overlap and connections between pathways.

In order to interrogate our proteomics data more deeply we analyzed our dataset with ingenuity pathway analysis (IPA). First we examined which canonical pathways are conserved between rat and human. Many cellular pathways are conserved between the two species including EIF2 signaling, mTOR signaling, Rho Family GTPases, and Fatty Acid oxidation (Fig. 4A). Additionally, dissection of pathways with relevance in inflammation and neuronal signaling and function demonstrates the presence of proteins specific to these functions in both species. In particular the CRCX4, P2Y, Andregeneric, and Axonal guidance pathways were well represented in the proteomes of both species (Fig. 4B–E). Mapping to diseases and functions also showed many core cellular functions as well as neuronal specific functions were well represented in both proteomes (Fig. 4F). Notably the top ranking sub-category within Nerveous System Development and Function was Neuritogenesis. We also examined the cannonical processes best represented by the top 100 most abundant proteins in humans and rats. Glycolysis and gluconeogenesis are the best represented pathways amongst these sets of proteins indicating the conservation of some core metabolic functions between rat and humans. Next we examined what pathways are represented in one species but not the other. Proteins only detected in human tissue could be bucketed into pathways involved in innate immunity such as Rig1-like receptors and activation of IRF pathways suggesting human and rat DRGs may respond differently to stimuli that interact with these pathways. On the other hand, rat proteins were bucketed into pathways such as choline and catecholamine biosynthesis (Fig. 5) suggesting some differences in endogenous metabolites between human and rat DRG. Proteins that are unique to rat best represented the neurological disease categories abnormal morphology of pyridimal neurons, abnormal morphology of somatic nerveous system, and abnormal morphology of the myelin sheeth. Whereas proteins that are unique to humans best represented the neurological disease categories cell death of granule cells, disruption of the blood brain barrier, and primary central nerveous system lymphoma. These analysis provide a guide to well conserved pathways that are more likely to respond similarly between rats and humans and pathways which are less likely to track closely between species. Of course, these analysis are based on protein detection and other factors must be considered as well such as post-translational modifications, lipid and metabolite differences, and dynamic intercellular interactions.

### Proteome Profiling Predicts MAP4K4 Inhibitor Efficacy in Neurite Outgrowth

**Figure 6:**
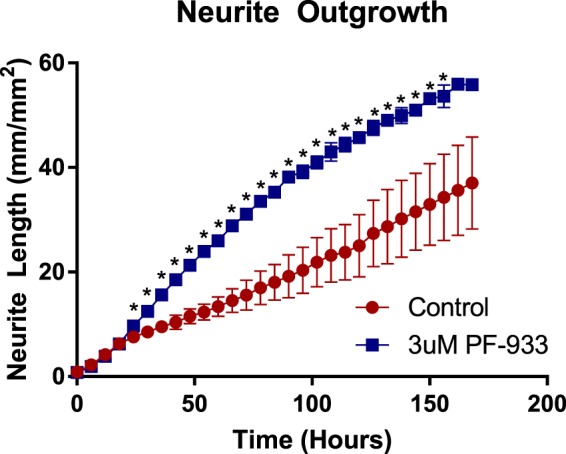
Neurite outgrowth in rat DRG is enhanced by treatment with MAP4K4 inhibitor. DRG were plated at 0 hours. They were treated with 3uM PF-933 at 20 hours. (*Student’s t-test, p-value < 0.05).

Ultimately, we hoped profiling both rat and human DRG would help us to identify mechanisms that were translatable between model systems and human. As mentioned above, we noted that Neuritogenesis was the most represented pathway in the category of Neuronal Development and Function by proteins shared between rat and human DRG. This led us to ask if neurite outgrowth is a conserved phenotype between human and rodent systems. Neurite outgrowth is sometimes used as a cellular proxy for pain^9,10^. Known examples of translatability do exist, particularly in PC12, and SH-SY5Y cell systems. However, there are notable differences between the transcriptome of human and murine nerveous system tissue and between the transcriptome of SH-SY5Y and DRG. There are also some assertions that iPSC or primary neuron cell systems are superior models to immortalized cell lines^22–24^. In principle, it would be most interesting if we could use our dataset to select a relatively unstudied mechanism of neurite outgrowth and predict translation to a DRG model. We noted that the kinase MAP4K4 was expressed in both human and rat DRG and is upstream of Rho GTPase signaling, which is strongly represented in our Ingenuity Pathway Analysis results^25,26^. Recently, MAP4K4 was shown to have a role in neuronal morphogenesis, in particular neurite outgrowth, in the human neuroblastoma derived SH-SY5Y cell line, but relative to some stimuli of neurite outgrowth MAP4K4 inhibition is relatively unstudied^27^. Our DRG proteomics data also indicated that MAP4K4’s substrates FARP1, ezrin, and moesin, which control cell morphology, are expressed in both rat and human DRG. So, based on our proteomics data we asked if MAP4K4 inhibitor treatment could induce neurite outgrowth in rat DRG^27,28^. We treated rat DRG with the MAP4K4 inhibitor PF-933 and found a clear increase in neurite length relative to DMSO beginning 5 hours after inhibitor treatment (Fig. 6). Additionally, this is the same dosage that was previously used for human SH-SY5Y. These data demonstrate the value of untargeted proteomics in tissue from model organisms and humans to identify species and tissue translatable mechanisms.

## Discussion

In total, we have identified more than 4,959 proteins from rat and human dorsal root ganglion and found that the DRG proteome is somewhat similar from one species to the other with some key differences in relative protein abundance and the proteins identified. Proof of principle studies with a MAP4K4 inhibitor suggests this data can be used to assist in predictions of translatability between rodents and humans. These experiments demonstrate that proteomics data across species can aid in the prediction of mechanisms or phenotypes that will translate from one species to another. Additional information, such as intercellular interactions, dynamic signaling changes, and interactions with surrounding tissues are also important for the translatability of mechanisms and phenotypes and are not captured in this study. Comprehensive proteomic profiling does allow key criteria, such as the abundance of a protein and it’s network of protein interaction partners or substrates, to be determined and used to predict translatability.

While there are well studied pathways involved in pain that are known to be translatable between species perhaps one of the best ways to prospectively apply the sorts of proteomic datasets we present here are in combination with other genomic or proteomic datasets. Often studies apply these technologies to globally measure changes between healthy and diseased states in model organisms with the intention of nominating new proteins with a role in pain in humans. For example, Zhang *et al*. used 2D-gel electrophoresis combined with MALDI-TOF to measure proteins whose abundance was changed by chronic compression of DRG in order to identify proteins with a role in pain^12^. Our data can then be applied to verify that these proteins are also detected in human DRG, which supports the notion that they could be translatable to a role in human pain. On the other hand, Komori *et al*. performed a similar study looking at proteins that changed in the case of spinal nerve injury^11^. Again, most proteins detected in this study were also detected in human DRG, but two, Guanine Deaminase and Spindlin-2B, were not. The absence or decreased abundance of these proteins in humans relative to model species may mean that these two proteins are relatively poor candidates for human translation.

The application of this sort of triaging to support or refute human translatability becomes more apparent as it is applied to larger datasets generated by more contemporary technologies. For instance, Rowette *et al*. used data-independent acquisition mass spectrometry to identify proteins whose abundance changed in chronic inflammatory and neuropathic pain^13^. One protein they nominated as having a potential role in pain is DPP4. We do not detect this protein in human DRG suggesting that the effect of DPP4 inhibition on pain in rodent models may not be translatable to man. Notably, to our knowledge, there are no published reports of clinical DPP4 inhibitors decreasing chronic pain in human clinical trials. The application of our dataset to genome wide measurements in model organisms is particularly relevant. For instance, Sapio *et al*. measured gene expression in rat DRGs and other neuronal tissues and predicted genes involved in neuropathy based on these measurements. However, comparison of this data to our human proteomics data shows there is no correlation between rat and human protein abundance of these genes (Spearman Correlation −0.10), which may raise questions regarding translatability of these genes as targets for pain in humans. We envision that this sort of strategy, in combination with other omics methods, will be used to overcome the translatability gap that exists between preclinical species and man. Ultimately, we anticipate these data will be used in further studies that seek to study mechanisms in DRG or translate results from model organisms to man.

## Methods

All methods were carried out in accordance with relevant guidelines and regulations.

### Rat DRG Collection

All animal studies were conducted in accord with the Guide for the Care and Use of Laboratory Animals (Institute of Laboratory Animal Resources, Commission on Life Sciences, National Research Council, 2011) and were approved by the Institutional Animal Care and Use Committee at MRL, Merck & Co. Rat Dorsal Root Ganglion were harvested as descriped^29^.

### Human DRG Collection, Donor information, and Permission

All studies were carried out in accordance with relevant guidelines and regulations. Experimental protocols were supported by Merck Research Laboratories. All human tissues were obtained from ANABIOS. All human tissues used for the study were obtained by legal consent from organ donors in the US. ANABIOS Corporation’s procurement network and includes only US based Organ Procurement Organizations and Hospitals. Policies for donor screening and consent are the ones established by the United Network for Organ Sharing (UNOS). Organizations supplying human tissues to ANABIOS follow the standards and procedures established by the US Centers for Disease Control (CDC) and are inspected biannually by the Department of Health and Human Services (DHHS). Tissue distribution is governed by internal IRB procedures and compliance with HIPAA regulations regarding patient privacy. All transfers of donor organs to ANABIOS are fully traceable and periodically reviewed by US Federal authorities.

Human DRGs were collected as previously described^30^.

### DRG Treatment with PF-933

Rat DRG neurons (Lonza, #R-DRG-505, lot# 140316) were plated in Neurobasal A media (Invitrogen, #12349), 2% B-27 (Invitrogen, #17504), 1x Penicillin/Streptomycin (Invitrogen, #15140), 1x L-Glutamine (Invitrogen, #35050–079) at the density of 2000 cells per well on Poly-L-Ornithyne (0.01% solution from Sigma Cat #P4957) and poly-D-lysine coated uClear (greiner-bio-one Cat #655946) 96 well plates. After overnight incubation at 37 °C and 5% CO2 cells were treated with 3 µM PF-933 (Sigma #PZ0272) or DMSO (Sigma #D5879). Cells were monitored with an IncuCyte ZOOM from Essen Biosciences. Changes in neurite length were monitored using IncuCyte NeuroTrack^TM^ analysis software.

### DRG sample preparation

DRGs were transferred to Navy RINO Bullet Homogenizer tubes (NextAdvance). DRGs were suspended in 100 uL cold 50 mM ammonium bicarbonate with HALT protease and phosphatase inhibitors with EDTA (Fisher Scientific). ProteaseMax was added to 0.1% w/v. DRGs were lysed in bullet homogenizer (NextAdvance) at power level 8 for 3 minutes at 4 °C. Supernatant was collected. To remaining tissue fragments, 100 uL 8 M urea in 50 mM ammonium bicarbonate was added. Samples were relysed at power level 8 for 3 minutes at 4 °C. Lysate was pooled from each sample. Lysate was spun down at 20,000 at 4 °C for 10 minutes. Supernatant was collected. A bicinchoninic acid colorimetric assay was performed for protein quantitation. 30 ug of protein from each sample was used for subsequent steps. Samples were reduced and alkylated with 10 mM TCEP and 15 mM iodoacetamide at 37 °C for 1 hour. DTT was added to a concentration of 15 mM to quench unreacted iodoacetamide. Urea was diluted to 1 M with 50 mM ammonium bicarbonate. Calcium chloride was added to a concentration of 1 mM. 1ug of Trypsin/lysC (promega) was added per sample. Samples were digested overnight at 37 °C with shaking.

The next day, samples were acidified with formic acid and desalted on TopTip C18 columns. Samples were dried down after desalting, resuspended in 20 uL 95% water, 5% acetonitrile, 0.1% formic acid and samples were analyzed by mass spectrometry.

### Mass Spectrometry

Samples were analyzed on an Orbitrap Fusion-Lumos. 2 uL, amounting to 3 ug, of each sample was injected. Each sample was injected three times. Samples were injected onto a 75 cm C18 EASY-Spray column (Thermo Fisher Scientific) and analyzed over a 4 hour gradient from 3–40% acetonitrile. 0.1% formic acid was used as a solvent modifier. Water was the co-solvent. The emitter tip temperature was set at 55 °C. MS1s were measured in the orbitrap. MS2s were measured in the LTQ with a top 20 method.

### Data Analysis

Data was analyzed in Maxquant version 1.6.01. Precursor ppm was set at 5 ppm. Peptide FDR was 1%. Data was searched against the uniprot rattus norvegicus or homo sapien databases. Peptides had two tryptic ends and a maximum of 3 missed cleavages. Identified proteins had >1 unique and razor peptide. Protein rank was determined by the relative abundance of a protein in its proteome. Sample runs from the same species were processed together and the match between runs feature was used. Data from technical replicates was averaged. Gene Ontology Analysis was performed using the STRING bioinformatics tool (https://string-db.org/). The datasets generated during this study are available from the corresponding author on reasonable request.

Ingenuity Pathway Analysis (IPA) was performed with Qiagen’s Ingenuity Pathway Analysis software, Version 43605602. IPA settings were restricted to Nervous System tissue, CNS Cell Lines, Immune Cell lines, Macrophage cancer cell lines, Neuroblastoma Cell lines. Benjamini-Hochberg corrected p-values were used for all IPA analyses.

## Electronic supplementary material

Supplementary Figure 1

Supplementary Table 1

Supplementary Table 2

Supplementary Table 3

Supplementary Table 4
