# Supplementary material for: Comparison of the Rat and Human Dorsal Root Ganglion Proteome
Source: Sci Rep. 2018 Sep 7;8:13469. doi: 10.1038/s41598-018-31189-9 (PMC6128859; doi:10.1038/s41598-018-31189-9)
Supplement: Supplementary file 1 — Supplementary Figure 1 [file 41598_2018_31189_MOESM1_ESM.pdf]

Comparison of the Rat and Human Dorsal Root Ganglion Proteome

Adam G. Schwaid[1*], Alicja Krasowka-Zoladek[2], An Chi[1], Ivan Cornella-Taracido[1,3]

1. MRL, Merck & Co., Inc., Boston, MA 02115 USA
2. MRL, Merck & Co., Inc., West Point, PA 19846 USA
3. Current Affiliation: Cedilla Therapeutics, Cambridge, MA 02139

Supplementary Figure

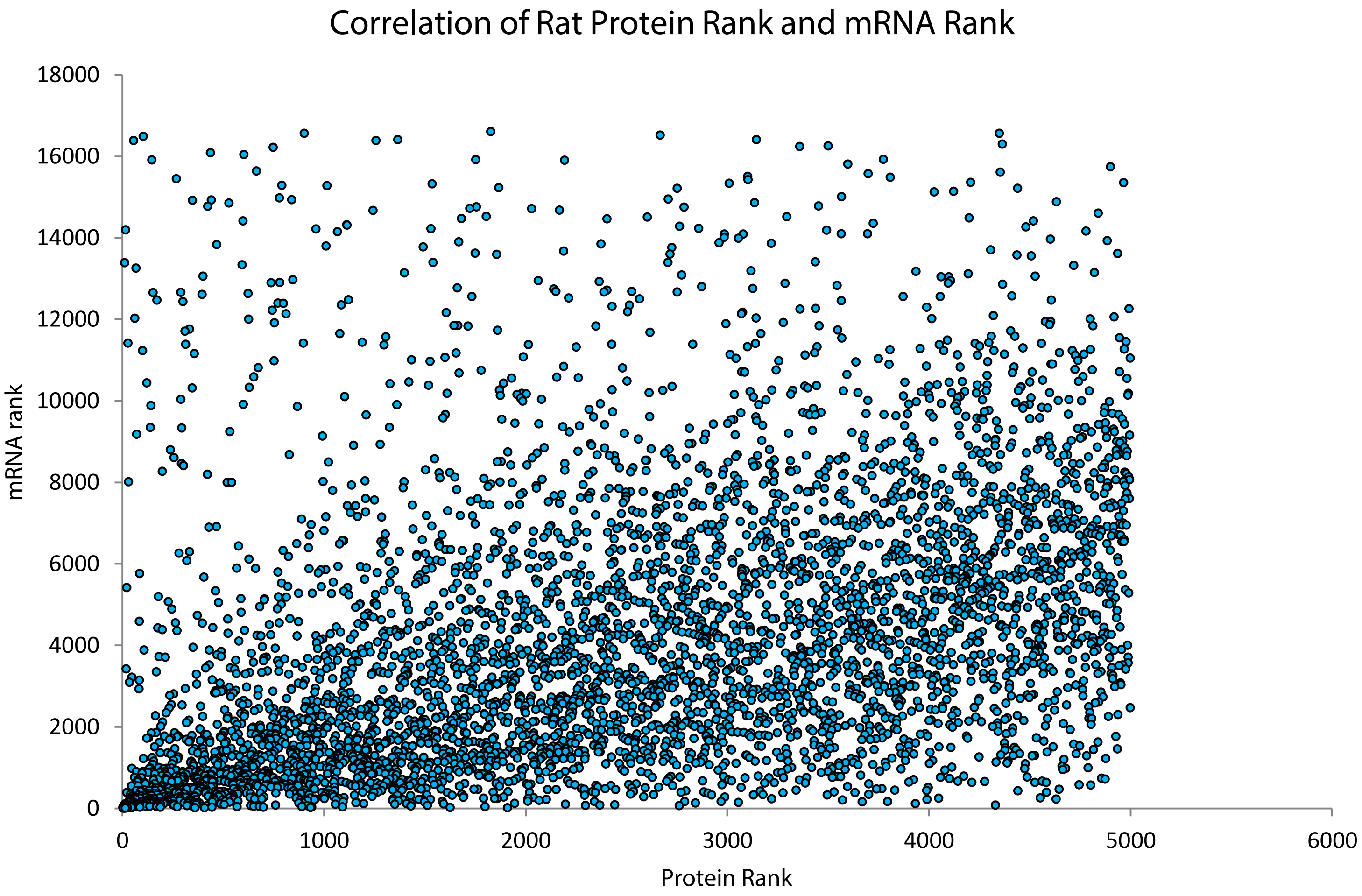

Supplementary Figure 1: Ranked mRNA abundance of genes measured in Sapio et al. plotted versus ranked protein abundance measured in this study.
